# Factors affecting the rates of incidental parathyroidectomy during thyroidectomy

**DOI:** 10.1308/rcsann.2024.0019

**Published:** 2024-03-06

**Authors:** HE Arslan, S Zeren, AC Yildirim, MF Ekici, O Arik, MC Algin

**Affiliations:** ^1^ Kutahya Health Sciences University, Turkey; ^2^ Eskisehir City Hospital, Turkey

**Keywords:** Incidental parathyroidectomy, Postoperative hypocalcaemia, Thyroidectomy, Resident, Surgeon

## Abstract

**Background:**

The most important factors affecting the development of postoperative hypocalcaemia (PH) include intraoperative trauma to the parathyroid glands, incidental parathyroidectomy (IP), and the surgeon's experience. In this study, we aimed to determine the incidence of IP, evaluate its effect on postoperative calcium levels and investigate the effect of surgeon experience and volume on IP incidence and postoperative calcium levels.

**Methods:**

This retrospective study included 645 patients who underwent thyroid surgery at the Department of General Surgery, Kütahya Health Sciences University between September 2016 and March 2020. All patients underwent surgery at a single clinic by general surgeons experienced in thyroid surgery and their residents (3–5 years).

**Results:**

Normal parathyroid glands were reported in 58 (8.9%) of 645 patients. In 5 (8.6%) of 58 patients the parathyroid gland was detected in the intrathyroidal region. PH developed in ten patients (17.2%) with incidental removal of the parathyroid glands. A statistically significant difference was found between the number of incidentally removed parathyroid glands and the development of hypocalcaemia (*p*<0.05). Normal parathyroid glands were reported in the pathology of 37 (7.9%) patients operated on by general surgeons and 22 (12.6%) patients operated on by their residents. PH developed in 39 (8.2%) patients operated on by general surgeons and in 8 (4.5%) patients operated on by their residents.

**Conclusions:**

We found that the complication rate during the resident training process was the same as that of experienced general surgeons. A thyroidectomy can be safely performed by senior residents during residential training.

## Introduction

Thyroidectomy is the most common endocrine surgery. The standardisation of thyroidectomy techniques and advances in perioperative management have led to significant reductions in overall mortality and morbidity in the 21st century. Currently, thyroidectomy has a complication rate of less than 5%, and is performed as a day surgery in selected cases.^1–3^ A complication is postoperative hypocalcaemia (PH). Defined as a serum calcium level lower than normal, PH requires calcium/vitamin D replacement therapy within 24h after surgery or when discharged from the hospital.^3^ PH remains the most common complication of thyroid surgery, with a prevalence ranging from 1.5% to 50%, depending on the definition of low serum calcium levels.

The incidence of transient hypocalcaemia after thyroid surgery has been reported to be 1.6–50%, and the incidence of permanent hypocalcaemia is between 1.5% and 4%.^4–6^ Symptoms of hypocalcaemia appear within the first 24–48h after surgery. PH may be associated with operative stress, calcitonin secretion due to surgery, vitamin D deficiency due to postoperative alkalosis resulting from hyperventilation triggered by postoperative pain, or haemodilution. The most important factors affecting the development of PH include intraoperative trauma to the parathyroid gland or vascular system, failure to identify the parathyroid gland during surgery, acute parathyroid insufficiency resulting from incidental parathyroidectomy (IP), and surgeon experience.

Hypocalcaemia resolves spontaneously in many patients, but if irreversible damage occurs in the parathyroid gland, hypocalcaemia may be permanent.^3,6,7^ IP has been defined in the literature as parathyroid tissue found in a postoperative specimen and is a relatively common finding in thyroid pathology reports, even in the hands of experienced endocrine surgeons, with an incidence of 2.9–31%. In the literature, it has been reported that 2.2–50% of incidentally excised parathyroid glands are identified as postoperative intrathyroidal, with 16.7–40% located intracapsularly and 15.7–81.1% located extracapsularly.^1,4–7^

Variations in the number and anatomical location of parathyroid glands increase the risk of IP. Various anatomical variants of the parathyroid glands exist, especially in the inferior parathyroid glands.^6,7^ There is no consensus on biochemical and clinical outcomes or on identifying predisposing factors and high-risk patients. The development of hypocalcaemia as a result of incidental excision of the parathyroid glands is not necessary, but may be followed by biochemical or clinical hypocalcaemia.^5,7^

In this study, we aimed to determine the incidence of IP in patients who have undergone thyroid surgery in our clinic, evaluate its effect on postoperative calcium levels, investigate the effect of surgeon experience and volume on IP incidence and postoperative calcium levels, and determine possible risk factors in the light of preoperative and intraoperative factors.

## Methods

This retrospective study included 645 patients who underwent thyroid surgery at the Department of General Surgery, Kütahya Health Sciences University between September 2016 and March 2020. Permission for the study was obtained from the Non-Interventional Clinical Research Ethics Committee of Kütahya Health Sciences University (Ethics Committee Decision No: 2021/11-35). The Declaration of Helsinki was adhered to and no patient's personal information was disclosed during the study.

Patients diagnosed with multinodular goitre or nodular goitre, patients diagnosed with Graves' disease, patients with thyroid cancer, male and female patients aged 19–84 years, and patients with American Society of Anesthesiologists scores of I, II and III were included in the study. Pregnant women, patients younger than 18 years of age, patients with a history of preoperative laryngeal surgery, patients with a history of radiotherapy to the neck region, patients detected to have preoperative parathyroid pathology, patients that are not preoperatively normocalcaemic and patients with stage 3 and above chronic kidney disease were not included in the study.

All patients underwent preoperative and postoperative vocal cord examinations using a flexible fibre optic laryngoscope by a specialist in the Otorhinolaryngology Clinic. All patients underwent surgery at a single clinic by general surgeons experienced in thyroid surgery and their residents (3–5 years experience). Preoperative hospital records, age, sex, comorbidities, laboratory results, preoperative calcium (mg/dl) and albumin (g/dl) levels, ultrasonography findings and ear, nose, and throat (ENT) consultations were collected retrospectively.

The type and duration of the operation, the person performing the operation, intraoperative nerve monitoring (IONM), the condition of the parathyroid glands, recurrent laryngeal nerve (RLN) visualisation and drain usage were examined from the archives and recorded. In addition, postoperative complications, pathology results, calcium values at the eighth hour and sixth month, and vocal cord movements detected during the ENT consultation were examined and recorded from archives and files.

We analysed retrospectively 684 patients who underwent thyroid surgery between 2016 and 2020; 16 patients with missing records, 22 without preoperative normocalcaemia and 1 with preoperative hypoparathyroidism were excluded from the study. A total of 645 patients with complete preoperative, intraoperative and postoperative records were included in this study.

The first calcium and albumin measurements in all patients were made at the eighth postoperative hour. Calcium values were corrected according to albumin values and recalculated. The corrected total blood calcium level was calculated using the following formula^8^:

$$\begin{array}{rl} & \mathrm{Total}\;\mathrm{blood}\;\mathrm{calcium}\;\mathrm{value}\;\mathrm{corrected}\;(\mathrm{mg}/\mathrm{dl})\\ & =0.8\times [\mathrm{normal}\;\mathrm{albumin}\;\mathrm{value}\;4\;g/\mathrm{dl}\\ & \;\text{–}\mathrm{patien}t^{\prime }s\;\mathrm{albumin}\;\mathrm{value}\;(g/\mathrm{dl})]\;\\ & \;+\mathrm{total}\;\mathrm{blood}\;\mathrm{calcium}\;\mathrm{value} \end{array}$$

Patients with or without symptoms whose corrected calcium levels (normal range 8.8–10.2mg/dl) were measured below 8.8mg/dl were considered to be hypocalcaemic. Patients with symptomatic hypocalcaemia were treated with oral calcium and vitamin D preparations, and intravenous calcium gluconate. All patients with hypocalcaemia were referred to the endocrinology clinic. Patients reported to have normal parathyroid tissue on pathology were evaluated for IP, and their number and anatomical location were recorded to evaluate their relationship with the development of hypocalcaemia.

### Surgical technique

All patients underwent surgery under general anaesthesia with endotracheal intubation. Sevoflurane was used as the main medication, and rocuronium was used as the muscle relaxant. Full-dose rocuronium and remifentanil were used in those operated on using the conventional direct visualisation technique, and low-dose rocuronium and remifentanil were used in those operated on using IONM.

Patients underwent surgery in a semi-sitting position with a classic Kocher incision. The subcutaneous tissue and platysma muscle were dissected intraoperatively. The strap muscles were dissected longitudinally and retracted laterally using retractors. First, the middle thyroid vein was sealed with an energy device, and the upper pole was dissected. In all patients, the upper poles were tied using 2/0 Vicryl sutures.

In all study patients, the RLN was dissected and visualised in the tracheoesophageal groove. All parathyroid glands were observed and preserved. Finally, the ligamentous structures between the trachea and thyroid gland were cut, and hemithyroidectomy was completed. The same steps and procedures were applied to other lobes, and a total thyroidectomy (TT) was completed. In a near-TT, one lobe was removed completely, whereas <1cm of thyroid tissue was left behind in the other lobe. In these cases, the RLN was visualised. In addition, negative-pressure Hemovac drains were placed in the operative area for the postoperative follow-up of all patients.

### Statistical analysis

Data obtained from the patients were recorded using the IBM SPSS (Statistical Package for the Social Sciences) Statistics 20 program in Excel. All statistical analyses were performed using this program. Descriptive measures (mean, standard deviation, etc) were used to analyse the quantitative (numerical) data of the variables. For the analysis of qualitative (categorical) variables, frequency tables with numbers and percentages were used. Crosstabs were created to show variations in the two qualitative variables relative to each other. The row percentages are provided in the crosstabs. The chi-square test was used to compare the proportions between two or more groups. The phi coefficient, Cramer's V coefficient, and normality coefficient values, which are the coefficients for unordered qualitative data, were obtained. Statistical significance was set at *p*<0.05. In other words, statistical evaluation was carried out with a 0.05 margin of error and a confidence level of 0.95.

## Results

Of 645 patients, 492 were female and 153 were male, and their mean age was 49.9±13.9 years (range 19–84 years). When the surgical procedures were examined, 552 (85%) bilateral TTs, 71 (11%) lobectomies, 12 (2%) bilateral sub-TTs and 10 (2%) reoperations (recurrence cases) were performed. In our study, 2,418 parathyroid glands (*n*=2,580) were at risk.

Normal parathyroid glands were reported in 58 (8.9%) of the 645 patients and PH developed in 47 (7.2%). PH developed in ten patients (17.2%) with incidental removal of the parathyroid glands. This rate was 6.3% (*n*=37) in the group that did not have normal parathyroid glands. This difference was statistically significant (*p*=0.003) (Table 1).

**Table 1:** Relationship between presence of incidentally removed parathyroid gland and development of hypocalcemia

		Developing PH	PH not developing	Total
Presence of incidentally removed parathyroid gland	Yes	10	49	59
		16.9%	83.1%	100.0%
	No	37	549	586
		6.3%	93.7%	100.0%
	Total	47	598	645
		7.3%	92.7%	100.0%
Chi-square (*χ*^2^)=8.974; *p*=0.003				

PH = postoperative hypocalcaemia.

Of the 645 patients, 73% (*n*=471) were operated on by general surgeons experienced in thyroid surgery and 27% (*n*=174) were operated on by residents under the supervision of specialists. PH developed in 39 (8.2%) patients operated on by general surgeons and in 8 (4.5%) patients operated on by residents under the supervision of specialists. There was no statistically significant difference between surgeries performed by residents and specialists in the development of hypocalcaemia (*p*=0.110) (Table 2).

**Table 2:** Relationship between development of PH and surgeon

		Surgeon		Total
		Resident	Specialist	
Result	Developing PH	166	432	598
		27.8%	72.2%	100.0%
	PH not developing	8	39	47
		17.0%	83.0%	100.0%
Total		174	471	645
		27.0%	73.0%	100.0%
Chi-square (*χ*^2^)=2.551; *p*=0.110				

PH = postoperative hypocalcaemia.

Normal parathyroid glands were reported in the pathology of 37 (7.9%) patients operated on by general surgeons and 22 (12.6%) patients operated on by residents under the supervision of specialists. Although the percentage of unintentionally removed parathyroid glands was higher among residents than among specialists, no statistically significant correlation was found between the presence of unintentionally removed parathyroid glands and the surgeon performing the surgery (*p*>0.05) (Table 3).

**Table 3:** Relationship between presence of an incidentally removed parathyroid gland and surgeon

		Surgeon		Total
		Resident	Specialist	
Presence of incidentally removed parathyroid gland	Yes	22	37	59
		37.3%	62.7%	100.0%
	No	152	434	586
		25.9%	74.1%	100.0%
Total		174	471	645
		27.0%	73.0%	100.0%
Chi-square (*χ*^2^)=3.505; *p*=0.061				

In 5 (8.6%) of the 58 patients who were reported to have normal parathyroid glands in their pathology, the parathyroid gland was detected in the intrathyroidal region.

A statistically significant difference was found between the number of incidentally removed parathyroid glands and the development of hypocalcaemia (*p*<0.05). While the rate of PH development was 6.3% (*n*=37) in the group in which the parathyroid gland was not excised incidentally, this rate was 14% (*n*=7) in the group in which one parathyroid gland was excised incidentally and 28.6% in the group in which two parathyroid glands were excised incidentally (*n*=2), and the rate of hypocalcaemia development was 100% in the only patient whose three parathyroid glands were incidentally excised (Table 4).

**Table 4:** Relationship between number of incidentally removed parathyroid glands and development of PH

		Developing PH	PH not developing	Total
Number of parathyroid glands incidentally removed	0	37	550	587
		6.3%	93.7%	100.0%
	1	7	43	50
		14.0%	86.0%	100.0%
	2	2	5	7
		28.6%	71.4%	100.0%
	3	1	0	1
		100.0%	0.0%	100.0%
	Total	47	598	645
		7.3%	92.7%	100.0%
Chi-square (*χ*^2^)=21.368 ; *p*=0.000				

PH = postoperative hypocalcaemia.

When the postoperative pathology reports were reviewed retrospectively, nodular hyperplasia (*n*=308) and papillary carcinoma (*n*=168) were predominant (Table 5). In addition, a statistically significant correlation was found between papillary carcinoma, nodular hyperplasia and IP (*p*=0.037) (Table 5).

**Table 5:** Relationship between postoperative pathological diagnosis of patients and presence of incidentally removed parathyroid glands

		Presence of incidentally removed parathyroid gland		Total
		Yes	No	
Postoperative Pathological Diagnosis	Adenomatous Hyperplasia	2	106	108
		1.9%	98.1%	100.0%
	Anaplastic Carcinoma	0	1	1
		0.0%	100.0%	100.0%
	Dyshormonogenetic Goiter	0	1	1
		0.0%	100.0%	100.0%
	Follicular Adenoma	0	17	17
		0.0%	100.0%	100.0%
	Follicular Carcinoma	1	1	2
		50.0%	50.0%	100.0%
	Granulomatous Inflammation	1	1	2
		50.0%	50.0%	100.0%
	Hurthle Cell Adenoma	0	4	4
		0.0%	100.0%	100.0%
	Hurthle Cell Carcinoma	0	5	5
		0.0%	100.0%	100.0%
	Lymphocytic Thyroiditis	2	22	24
		8.3%	91.7%	100.0%
	Medullary Carcinoma	0	4	4
		0.0%	100.0%	100.0%
	NIFTP	0	1	1
		0.0%	100.0%	100.0%
	Nodular Hyperplasia	38	270	308
		12.3%	87.7%	100.0%
	Papillary Carcinoma	15	153	168
		8.9%	91.1%	100.0%
	Total	59	586	645
		9.1%	90.9%	100.0%
Chi-square (χ^2^)=27.793; *p*=0.037				

NIFTP = noninvasive follicular thyroid neoplasm with papillary-like nuclear features

## Discussion

This study compared the presence, localisation and number of IP in the postoperative pathology reports of patients who underwent thyroid surgery by general surgeons and resident physicians under the supervision of experienced surgeons in a centre, and their effects on PH.

To the best of our knowledge, this is the first study to compare IP rates between senior resident physicians and experienced surgeons at the same centre. Our findings show that IP is common, with a rate of 7.9%, even among experienced surgeons. Since 8.6% of incidentally removed glands are intrathyroidal, it is not possible to completely avoid IP despite careful dissection and good anatomical knowledge. In this study, papillary carcinoma and nodular hyperplasia were identified as possible risk factors for IP, and the presence and number of incidental parathyroidectomies were associated with the development of PH. Evaluating these risk factors during the surgery will increase surgeons' awareness of IP and minimise the number of other preventable IP cases.

IP rates in the literature vary between 2.3% and 35.5%.^9–21^ Our data show that IP occurred at a rate of 8.9%. This result is consistent with rates reported in the literature. Although variable locations and numbers of parathyroid glands make intraoperative identification difficult and increase the risk of IP, several authors recommend careful examination of the specimen for the presence of parathyroid glands and autotransplantation into the adjacent sternocleidomastoid muscle.^9,21^ In a cadaver dissection of 942 individuals, a fifth parathyroid gland was found in 5% of cases and three parathyroid glands (instead of four) in 2% of cases.^22^

The intrathyroidal location of parathyroid glands is often an inevitable cause of IP. This condition, which has been reported in 0.2% of autopsy series,^23,24^ has been reported in 3–52.9% of cases.^10,11,13,15,17,20,21,25–28^ This higher incidence and wide range in the literature can be explained by thyroid malignancies, which could potentially lead pathologists to examine the thyroid tissue in more detail than in cadaver studies.^29^ In the present study, the rate was 8.6%.

Although the relationship between PH and IP has not been determined clearly in the literature,^5,13,30,31^ the incidence of PH in the IP group was approximately three times higher than that in the non-IP group in our study (16.9% vs 6.3%, *p*<0.003). Many studies investigating IP reported no statistically significant relationship between IP and postoperative biochemical hypocalcaemia.^29,32–34^ In a prospective study of 207 patients who underwent thyroid surgery, Youssef *et al* found no significant difference in the incidence of postoperative biochemical hypocalcaemia between patients with and without IP (7.7% vs 8.8%, *p*=0.55).^34^ The latter study involved a series of patients with high rates of lobectomy and near-TT (40.1%). In contrast, recent studies involving multiple cases of TT and patients with thyroid cancer, in whom all four glands were at risk, reported that patients with IP were more likely to develop postoperative biochemical hypocalcaemia.^13,17,20,25,26,35^

Several studies have concluded that thyroid malignancies increase the risk of IP.^12,14,28,36^ However, in other studies, thyroid malignancies reduced the risk of IP.^9,12,13,31^ In this study, according to univariate analysis, the incidence of thyroid malignancy and nodular hyperplasia, especially papillary carcinoma, was significantly higher in patients with IP. In our study, malignancy was found as the last pathology in 180 patients (27.9%). The high incidence of malignancy may indicate that it is an additional risk factor for IP. These results can be explained by the fact that patients with suspected thyroid malignancies can be treated with TT, reoperation for complete resection, or concomitant cervical lymph node dissection (CLND). Therefore, we believe that careful monitoring during thyroidectomy in patients with a preliminary diagnosis of papillary carcinoma and nodular hyperplasia may reduce the risk of IP.

In our study, the number of incidentally resected parathyroid glands was associated with the risk of hypocalcaemia. Patients with two resected parathyroid glands had a significantly higher risk of hypocalcaemia than those with only one gland resected incidentally (28.6% vs 14%, *p*<0.01). Furthermore, patients with three incidentally resected parathyroid glands had a significantly higher incidence of hypocalcaemia than those with two incidentally resected parathyroid glands (100% vs 28.6%, *p*<0.01). This result is consistent with those of other recent publications.^19,37,38^ Lorente-Poch *et al* published a prospective study of incidental parathyroidectomies.^38^ In the present study, the prevalence of hypocalcaemia and persistent hypoparathyroidism was associated with the number of parathyroid glands left in place. In addition, studies have reported that removing one or two glands does not significantly affect PH.^11,26,39^

Several studies have reported that TT is a risk factor for IP.^13,17,20,26,28^ In our study, 85.58% of the patients underwent TT, and no significant difference was found between the groups in terms of the surgical method used for IP (*p*=0.576). Our results are consistent with those of previous studies reporting that TT does not increase the IP rate.^9,11,12,14,21^ In this case, a statistically accurate analysis could not be performed because of the reduced use of other surgical methods.

Few articles in the literature discuss the effect of surgeon volume and experience on the incidence of IP and the development of PH. When the data of Lin *et al* and Manouras *et al* were analysed,^13,40^ there was no significant difference in the incidence of IP based on surgeon volume. In a retrospective study by Barrios *et al*,^41^ in which 1,114 thyroidectomies were examined, a higher case volume was associated with a lower incidence of unintentional parathyroidectomy for both thyroidectomy and CLND.

In our study, surgeon volume and experience were not associated with the incidence of incidentally removed parathyroid glands or PH. PH developed in 39 (8.2%) patients operated on by general surgeons and in 8 (4.5%) patients operated on by senior residents under the guidance of specialists. There was no statistically significant difference between the residents and specialists performing the surgery in terms of the development of hypocalcaemia (*p*=0.11). Normal parathyroid glands were reported in the pathology of 37 (7.9%) patients operated on by general surgeons and 22 (12.6%) patients operated on by residents. Although the percentage of unintentionally removed parathyroid glands was higher among residents than among specialists, no statistically significant correlation was found between the presence of unintentionally removed parathyroid glands and the surgeon performing the surgery (*p*>0.05).

We believe that the complication rate during the resident training process was the same as that of the specialists. However, patients managed by an experienced surgeon may have been more complicated cases, such as locally advanced thyroid malignancy, massive goitre, substernal goitre, and recurrent surgery.

### Study limitations

This study has some limitations. The first limitation is it is retrospective in design. Second, when the surgical records were examined, most surgeons did not document the number and localisation of the parathyroid glands that they identified and preserved perioperatively. Therefore, the relationship between the parathyroid definition and IP could not be evaluated. However, the assessment of the absence or presence of parathyroid glands identified during surgery was not objective and may differ between surgeons and studies. Third, the study was conducted at a single centre, which limits the generalisability of the statistics; however, the strength of our study is that the number of patients was sufficient for a single centre. Fourth, central CLND is the risk factor for IP reported most commonly in the literature.^21,36^ In our study, only one patient underwent CLND; therefore, the relationship between CLND and IP could not be evaluated.

Finally, we did not evaluate the effects of IP on the development of postoperative hypoparathyroidism. In addition, the lack of long-term follow-up of these patients did not allow us to identify the long-term sequelae of IP, which is a significant disadvantage.

## Conclusions

Because most parathyroid glands are extracapsular, the incidence of IP can be reduced by careful and meticulous dissection and strict adherence to anatomical and surgical principles. Of the incidentally excised parathyroid glands, 8.6% were classified as postoperative intrathyroid glands. The inability to perform autotransplantation in these cases indicates that complete elimination of the IP is difficult with the current surgical techniques. Papillary carcinoma and nodular hyperplasia have been identified as potential risk factors for IP. The presence and number of incidental parathyroidectomies were associated with the development of PH.

Age, sex and surgical technique were not identified as risk factors for IP. Surgeons should also consider anatomical variations in the parathyroid glands to avoid mechanical and thermal injury, devascularisation or resection of the parathyroid tissue. Patients should be warned about the possibility of accidental removal of the parathyroid gland during the consent process, as they can review the operative notes. These results suggest that investigating techniques aimed at identifying parathyroid glands intraoperatively may reduce the prevalence of this complication. We found that the complication rate during the resident training process was the same as that of experienced general surgeons. A thyroidectomy can be performed safely by senior residents during residential training.

## References

[1] Karadeniz E, Akcay MN. Risk factors of incidental parathyroidectomy and its relationship with hypocalcemia after thyroidectomy: a retrospective study. *Cureus* 2019; **11**: e5920. 31788378 10.7759/cureus.5920 PMC6857829

[2] Khairy GA, Al-Saif A. Incidental parathyroidectomy during thyroid resection: incidence, risk factors, and outcome. *Ann Saudi Med* 2011; **31**: 274–278. 21623057 10.4103/0256-4947.81545 PMC3119968

[3] Neagoe RM, Cvasciuc IT, Muresan M *et al.* Incidental parathyroidectomy during thyroid surgery - risk, prevention and controversies; an evidence-based review. *Acta Endocrinol (Buchar)* 2017; **13**: 467–475. 31149218 10.4183/aeb.2017.467 PMC6516554

[4] Du W, Fang Q, Zhang X *et al.* Unintentional parathyroidectomy during total thyroidectomy surgery: a single surgeon's experience. *Medicine (Baltimore)* 2017; **96**: e6411. 28296787 10.1097/MD.0000000000006411 PMC5369942

[5] Manatakis DK, Balalis D, Soulou VN *et al.* Incidental parathyroidectomy during total thyroidectomy: risk factors and consequences. *Int J Endocrinol* 2016; **2016**: 7825305. 27635137 10.1155/2016/7825305 PMC5007309

[6] Ozemir IA, Buldanli MZ, Yener O *et al.* Factors affecting postoperative hypocalcemia after thyroid surgery: importance of incidental parathyroidectomy. *North Clin Istanb* 2016; **3**: 9–14. 28058379 10.14744/nci.2016.48802 PMC5175085

[7] Bai B, Chen Z, Chen W. Risk factors and outcomes of incidental parathyroidectomy in thyroidectomy: a systematic review and meta-analysis. *PLoS ONE* 2018; **13**: e0207088. 30412639 10.1371/journal.pone.0207088 PMC6226183

[8] Seo ST, Chang JW, Jin J *et al.* Transient and permanent hypocalcemia after total thyroidectomy: early predictive factors and long-term follow-up results. *Surgery* 2015; **158**: 1492–1499. 26144879 10.1016/j.surg.2015.04.041

[9] Alraddadi T, Aldhahri S, Almayouf M *et al.* Risk factors of incidental parathyroidecomy in thyroid surgery. *Cureus* 2019; **11**: e6517. 32025436 10.7759/cureus.6517 PMC6988722

[10] Andre N, Pascual C, Baert M *et al.* Impact of incidental parathyroidectomy and mediastinal-recurrent cellular and lymph-node dissection on parathyroid function after total thyroidectomy. *Eur Ann Otorhinolaryngol Head Neck Dis* 2020; **137**: 107–110. 31959572 10.1016/j.anorl.2020.01.001

[11] Ataş H, Akkurt G, Saylam B *et al.* Central neck dissection is an independent risk factor for incidental parathyroidectomy. *Acta Chir Belg* 2021; **121**: 36–41. 32996827 10.1080/00015458.2020.1828677

[12] Hone RW, Tikka T, Kaleva AI *et al.* Analysis of the incidence and factors predictive of inadvertent parathyroidectomy during thyroid surgery. *J Laryngol Otol* 2016; **130**: 669–673. 27282361 10.1017/S0022215116008136

[13] Lin YS, Hsueh C, Wu HY *et al.* Incidental parathyroidectomy during thyroidectomy increases the risk of postoperative hypocalcemia. *Laryngoscope* 2017; **127**: 2194–2200. 28121013 10.1002/lary.26448

[14] McGoldrick DM, Majeed M, Achakzai AA *et al.* Inadvertent parathyroidectomy during thyroid surgery. *Ir J Med Sci* 2017; **186**: 1019–1022. 28155099 10.1007/s11845-017-1560-9

[15] Ozden S, Erdogan A, Simsek B *et al.* Clinical course of incidental parathyroidectomy: single center experience. *Auris Nasus Larynx* 2018; **45**: 574–577. 28807528 10.1016/j.anl.2017.07.019

[16] Ponce de Leon-Ballesteros G, Velazquez-Fernandez D, Hernandez-Calderon FJ *et al.* Hypoparathyroidism after total thyroidectomy: importance of the intraoperative management of the parathyroid glands. *World J Surg* 2019; **43**: 1728–1735. 30919027 10.1007/s00268-019-04987-z

[17] Prazenica P, O'Driscoll K, Holy R. Incidental parathyroidectomy during thyroid surgery using capsular dissection technique. *Otolaryngol Head Neck Surg* 2014; **150**: 754–761. 24496742 10.1177/0194599814521365

[18] Sahyouni G, Osterbauer B, Park S *et al.* Rate of incidental parathyroidectomy in a pediatric population. *OTO Open* 2021; **5**: 2473974X211059070. 10.1177/2473974X211059070 PMC8597068 34805719

[19] Vasileiadis I, Charitoudis G, Vasileiadis D *et al.* Clinicopathological characteristics of incidental parathyroidectomy after total thyroidectomy: the effect on hypocalcemia. A retrospective cohort study. *Int J Surg* 2018; **55**: 167–174. 29864531 10.1016/j.ijsu.2018.05.737

[20] Yazici P, Bozkurt E, Citgez B *et al.* Incidental parathyroidectomy as a cause of postoperative hypocalcemia after thyroid surgery: reality or illusion? *Minerva Chir* 2014; **69**: 315–320. 25242004

[21] Zheng J, Song H, Cai S *et al.* Evaluation of clinical significance and risk factors of incidental parathyroidectomy due to thyroidectomy: a single-center retrospective clinical study. *Medicine (Baltimore)* 2017; **96**: e8175. 28953673 10.1097/MD.0000000000008175 PMC5626316

[22] Lappas D, Noussios G, Anagnostis P *et al.* Location, number and morphology of parathyroid glands: results from a large anatomical series. *Anat Sci Int* 2012; **87**: 160–164. 22689148 10.1007/s12565-012-0142-1

[23] Akerstrom G, Malmaeus J, Bergstrom R. Surgical anatomy of human parathyroid glands. *Surgery* 1984; **95**: 14–21. 6691181

[24] Akerstrom G, Rudberg C, Grimelius L *et al.* Causes of failed primary exploration and technical aspects of re-operation in primary hyperparathyroidism. *World J Surg* 1992; **16**: 562–568; discussion 568–569. 1413826 10.1007/BF02067321

[25] Applewhite MK, White MG, Xiong M *et al.* Incidence, risk factors, and clinical outcomes of incidental parathyroidectomy during thyroid surgery. *Ann Surg Oncol* 2016; **23**: 4310–4315. 27541813 10.1245/s10434-016-5439-1

[26] Doulaptsi M, Ierodiakonou D, Prokopakis E *et al.* Effect of incidental parathyroidectomy on postoperative calcium levels after total thyroidectomy. *Hippokratia* 2020; **24**: 72–76. 33488055 PMC7811874

[27] Song CM, Jung JH, Ji YB *et al.* Relationship between hypoparathyroidism and the number of parathyroid glands preserved during thyroidectomy. *World J Surg Oncol* 2014; **12**: 200. 25000948 10.1186/1477-7819-12-200 PMC4105163

[28] Zhou HY, He JC, McHenry CR. Inadvertent parathyroidectomy: incidence, risk factors, and outcomes. *J Surg Res* 2016; **205**: 70–75. 27621001 10.1016/j.jss.2016.06.019

[29] Del Rio P, De Simone B, Viani L *et al.* Unintentional parathyroidectomy and postoperative hypocalcaemia. Conventional thyroidectomy versus miniinvasive thyroidectomy. *Ann Ital Chir* 2014; **85**: 470–473. 25599957

[30] Erbil Y, Barbaros U, Ozbey N *et al.* Risk factors of incidental parathyroidectomy after thyroidectomy for benign thyroid disorders. *Int J Surg* 2009; **7**: 58–61. 19027373 10.1016/j.ijsu.2008.10.012

[31] Gourgiotis S, Moustafellos P, Dimopoulos N *et al.* Inadvertent parathyroidectomy during thyroid surgery: the incidence of a complication of thyroidectomy. *Langenbecks Arch Surg* 2006; **391**: 557–560. 16951969 10.1007/s00423-006-0079-8

[32] Irkorucu O, Tascilar O, Cakmak GK *et al.* Inadvertent parathyroidectomy and temporary hypocalcemia: an adverse natural outcome or a true complication during thyroidectomy? *Endocr Regul* 2007; **41**: 143–148. 18257650

[33] Ondik MP, McGinn J, Ruggiero F *et al.* Unintentional parathyroidectomy and hypoparathyroidism in secondary central compartment surgery for thyroid cancer. *Head Neck* 2010; **32**: 462–466. 19780055 10.1002/hed.21205

[34] Youssef T, Gaballah G, Abd-Elaal E *et al.* Assessment of risk factors of incidental parathyroidectomy during thyroid surgery: a prospective study. *Int J Surg* 2010; **8**: 207–211. 20060940 10.1016/j.ijsu.2009.12.008

[35] Philips R, Nulty P, Seim N *et al.* Predicting transient hypocalcemia in patients with unplanned parathyroidectomy after thyroidectomy. *Am J Otolaryngol* 2019; **40**: 504–508. 31027850 10.1016/j.amjoto.2019.04.006 PMC9017535

[36] Sitges-Serra A, Gallego-Otaegui L, Suarez S *et al.* Inadvertent parathyroidectomy during total thyroidectomy and central neck dissection for papillary thyroid carcinoma. *Surgery* 2017; **161**: 712–719. 27743717 10.1016/j.surg.2016.08.021

[37] Lang BH, Chan DT, Chow FC. Visualizing fewer parathyroid glands may be associated with lower hypoparathyroidism following total thyroidectomy. *Langenbecks Arch Surg* 2016; **401**: 231–238. 26892668 10.1007/s00423-016-1386-3

[38] Lorente-Poch L, Sancho JJ, Ruiz S *et al.* Importance of in situ preservation of parathyroid glands during total thyroidectomy. *Br J Surg* 2015; **102**: 359–367. 25605285 10.1002/bjs.9676

[39] Spaziani E, Di Filippo AR, Di Cristofano C *et al.* Incidental parathyroidectomy during total thyroidectomy as a possible risk factor of hypocalcemia. experience of a single center and review of literature. *Acta Endocrinol (Buchar)* 2021; **17**: 207–211. 34925569 10.4183/aeb.2021.207 PMC8665250

[40] Manouras A, Markogiannakis H, Lagoudianakis E *et al.* Unintentional parathyroidectomy during total thyroidectomy. *Head Neck* 2008; **30**: 497–502. 18059011 10.1002/hed.20728

[41] Barrios L, Shafqat I, Alam U *et al.* Incidental parathyroidectomy in thyroidectomy and central neck dissection. *Surgery* 2021; **169**: 1145–1151. 33446359 10.1016/j.surg.2020.11.023

